# Down syndrome: A curative prospect?

**DOI:** 10.3934/Neuroscience.2020012

**Published:** 2020-06-22

**Authors:** Jean A. Rondal

**Affiliations:** University of Liège, Cognitive Sciences, Building 32, Sart Tilman, Liège 4000, Belgium

**Keywords:** Down syndrome, genetic therapy, early intervention, cognitive pharmacotherapy, Alzheimer disease

## Abstract

Experimental work regarding corrective actions on chromosomes and genes, and control of gene products is yielding promising results. It opens the way to advances in dealing with the etiological aspects of Down syndrome and may lead to important changes in the life of individuals affected with this condition. A small number of molecules are being investigated in pharmacological research that may have positive effects on intellectual functioning. Studies of the pathological consequences of the amyloid cascade and the TAU pathology in the etiology of Alzheimer disease (AD), which is more frequent and occuring earlier in life in persons with Down syndrome (DS), are presented. The search for biological markers of AD and ways for constrasting its early manifestations are also discussed.

## 1. Introduction

The prevalence rate of intellectual disability in the general population is estimated to be between 1 and 3%. Chromosome abnormalities (numerical as well as structural) are responsible for up to 28% of intellectual disabilities [1], among which aneuploidies are well represented. DS is the most frequent autosomal trisomy (chromosome 21–C21) occurring naturally in approximately 1 in 700 live births.

C21 actually is the smallest of human chromosomes. It should bear number 22 as chromosomes are numbered in size order and there are 22 pairs of autosomes plus one pair of sex chromosomes, XX for females and XY for males. The misplacement of C21 in chromosome order of magnitude goes back to an early time in molecular genetics where microscopes did not have the discriminative power that they acquired later, and the numerical mistake has not been corrected. Human C21, technically called Hsa21, harbors around 250 protein-coding genes (the precise number varying depending on genome annotation) and between 165 and 404 non-coding RNA (ribonucleic acid) genes regulating gene expression.

Overexpression of proteins linked to gene triplication determines a constellation of abnormalities involving heart, nervous system and gastro-intestinal tract. Impaired brain development causing structural anomalies and decreased volumes of frontal and temporal cortices, hippocampus, cerebellum, and brain stem, is typically observed. Anomalies of neural connectivity are the rule. However, almost every aspect of the DS phenotype is subject to an important degree of interindividual variability due to its polygenic nature and interactions with environmental factors [2].

DS appears in several forms. The most common one (95% of cases) is standard trisomy 21-TR21- (karyotype 47 + 21). It is characterized by a triplication of Hsa21 in every cell of the body. In mosaic TR21 (1 to 2% of the cases), only a portion of the cells carries one extra Hsa21. The proportion depends on the moment when the triplication of Hsa21 occurs (first, second, or third cell division). In Robertsonian (centric fusion; nonreciprocal) translocations, the participating chromosomes (pairs 13, 14, 15, 21, or 22) break at their centromeres and the long arms (q segments) fuse to form a single large chromosome with a single centromere. The short segments (p) are usually lost.

Robertsonian translocations involving C21 are: C21 with C21, C14, or more rarely C15: formulae 46 t (21; 21) + 21, 46 t (14; 21) + 21, and 46 t (15; 21) + 21, respectively (globally 3% of the cases). Partial TR21 (less than 1% of the cases) witnesses only a segment of Hsa21 being triplicated.

DS maps to a region on the long arm of Hsa21 covering an area of 37 to 44 megabytes corresponding to band 21q22 and containing around 225 genes [3]. Ait Yahya-Graison et al. [4] supply a figure of 200–250 genes between 21q21 and 21q22.3. A smaller region in Hsa21, labelled DSCR (Down Syndrome Critical Region), involving bands 21q21.1 and 21q22.2, which includes about 50 protein-coding genes and a larger number of non-coding elements, may arguably harbor most of the critical determinants of the phenotype of the condition [5].

Other analyses suggest that there may be several critical regions for different phenotypes and not just one region for the whole phenotype. For example, heart defects in DS map to a particular area of 5.2 megabytes within Hsa21 [6],[2]. Other specific sub-regions of Hsa21 are associated with hypotonia [3] and with one form of acute leukemia (megakaryoblastic) accounting for about 20% of the cases of leukemia in children with DS [7].

It is also possible that the overexpression of a number of genes on Hsa21 in TR21 leads to a genetic imbalance that deregulates the expression of other genes in other regions of Hsa21 or even in the entire genome [8]. The expression levels of genes located outside of Hsa21 may also be altered as some of the Hsa21 genes have a regulatory role extending beyond this chromosome [9].

Pelleri et al. [10] and Pelleri et al. [11] published the results of a reanalysis of 132 cases of partial segmental TR21. The main result is that there is one highly restricted region in DSCR, which they dubbed HR (highly restricted)-DSCR, of only 34 kilobases. It is located in the distal part of the 21q22.13 sub-band. Duplication of this region is shared by all subjects with DS but is absent in people without DS. A caveat is that this region contains no known gene. As to the identification of the genetic determinants located in HR-DSCR, the authors speculate that unknown microRNAs (miRNAs) in this region could be involved in DS pathology with the capability to regulate a large number of protein-coding genes. As a result, the HR-DSCR could carry longer-range interactions with other chromosomes.

The cause of standard TR21 is chromosomal nondisjunction mostly during meiosis I in the maternal egg. Paternal nondisjunction occurs during meiosis II in spermatogenesis. Translocations involving Hsa21 may occur *de novo* during syngamy or be inherited from parental genotypes (in about one quarter of the cases). Although these parents as heterozygous carriers are phenotypically normal (they have no genetic material in excess or deficit and their translocation is equilibrated), they have a 10% risk of having a child with DS if the mother carries the translocation and 2.5% risk if the father is the carrier [12].

This paper analyses ongoing work in genetics, epigenetics, and cognitive pharmacology relevant to the neurobiology of DS. In spite of a large number of conceptual and methodological limitations of these studies, the preliminary character of most of the findings reported, and the biologcal gaps between animal (murine) and human cognition, it would seem that this trend of research holds a potential for improving the neurobiology and possibly behavioral aspects of people affected with DS.

Neurocognitive development and functioning in persons with DS have been the object of much research worldwide over the last 75 years. There is no need to represent all or parts of this huge literature in the present context nor to propose a detailed analysis of what is known of the cognitive processes and limitations of people with DS. (See, for example, [13]; for analyses of the language difficulties of persons with DS, one may consult [14],[15]).

## 2. Genetic and epigenetic approaches

Chromosome and gene correction and reduction of excess protein production contributed by the triplication of a number of genes along the long arm of Hsa21 are the objects of ambitious experimental attempts.

### 2.1. Chromosome correction

At least three different techniques were reported to have met success in removing one extra Hsa21 in trisomic cells.

Takahashi et al. [16] demonstrated that induced pluripotent stem cells (iPSCs) can be generated from adult human dermal fibroblasts through genetic engineering using transcription factors. Pluripotent stem cells are capable of self-renewing and differentiating into a limited set of specialized cells in the body such as blood, liver, heart, or brain cells, but not all types of cells as is the case for embryonic or so-called omnipotent or multipotent stem cells originating from the inner mass of the embryonic blastocyst.

On this basis, Li et al. [17] generated iPSCs from fibroblasts obtained from adults with DS. They introduced a *TKNEO* fusion transgene carried by a modified adenovirus at the locus 21q21.3 of the gene *APP* (amyloid-bêta precursor protein) into one copy of Hsa21. This gene was chosen due to its location on Hsa21 and high expression in iPSCs.The operation resulted in spontaneous loss of an entire copy of Hsa21 in a large majority of clones while point mutations, epigenetic silencing, and *TKNEO* deletions occurred at lower frequencies in the five experiments undertaken. No damage to other chromosomes was observed.

Disomic cells proliferated faster in a co-culture than their trisomic counterparts doubling their population on average in about 37 ± 0.7 hrs against 45 ± 0.09 hrs for trisomic counterparts. The authors suggest that iPSCs offer a promising way to study human trisomy because they can be derived from the somatic cells of individuals with trisomy. Their approach could also be used to eliminate unwanted trisomies arising in stem cell cultures.

Jiang et al. [18] took advantage of a natural phenomenon to correct TR21. Nature has evolved a mechanism to compensate for the difference in dosage of X-linked gene copies between mammalian females and males. In humans, the formulae for the sex chromosomes are XY for males and XX for females. However, the Y chromosome is much smaller than its X counterpart. It contains only a few dozen genes compared to about 3000 for the X. Natural X dosage reduction in females is driven by a particular large non-coding RNA, named *XIST* (for X-inactive specific transcript) produced exclusively from the inactive X chromosome. This RNA inactivates the DNA (deoxyribonucleic acid) of this chromosome through methylation and chromatin modification turning it into a Barr body.

Jiang et al. [18] reprogrammed fibroblasts obtained from males with DS into iPSCs. They inserted a transgene *XIST* at locus 21q22 of the gene *DYRK1A* in one of the three Hsas21. This silenced this chromosome in 85% of the clones treated. Silencing of a dozen genes on the inactivated Hsa21 was confirmed. No alterations of the other chromosomes were observed. A few sub-clones remained in the 245 colonies of cells treated showing either one Hsa21 fused with the *XIST* RNA, two Hsas21 in the same state, or the three Hsas21 fused with the *XIST* RNA.

As in the experiment of Li et al. [17], disomic cells exhibited a capacity for *in vitro* proliferation above trisomic counterparts.

Another RNA in mammalian females, antagonist of *XIST*, named *TSIX* (anagram for *XIST*), has been identified. *XIST* and *TSIX* neutralize each other on the X chromosome that remains active, whereas the expression of *TSIX* is stopped on the inactivated X chromosome.

In the experiment of Jiang et al. [18], the fibroblasts were obtained from males with DS. It is not known what would happen in the same situation using fibroblasts from females with DS. Applying the above *XIST* dosage compensation technique, one could end up with some cells in the colonies treated exhibiting one Hsa21 and one X chromosome inactivated, and others where one Hsa21 and the two X chromosomes would be inactivated.

Natural silencing of one chromosome X in females is never complete and the choice of the genes remaining active is random. However, Jiang et al. [18] reported that the global expressivity of the two active Hsa21 was reduced by 20, 15, and 19%, respectively in the three clones tested, which is close to the 22% usually observed in disomic iPSCs that lack the third Hsa21 altogether. This suggests that the *XIST* RNA inserted in the extra Hsa21 covers key regions of this chromosome actually preventing transcription factors from reading the sequence of nucleic acids. *XIST* appears to induce a robust dosage compensation of most Hsa genes overexpressed in TR21.

Amano et al. [19] normalized the karyotypes in a culture of mouse embryonic stem cells engineered to become aneuploid or polyploid, using a biologic made of a mammalian-specific gene, *ZSCAN4* (zinc finger and scan domain) containing 4 transcription factors regularly expressed in pre-implantation embryos and occasionally in stem cells, which were encoded for delivery in a synthetic messenger RNA (mRNA) and Sendai virus vector.

They tested this biologic on iPSCs generated from fibroblasts obtained from individuals with DS and carrying standard TR21. Within a few weeks, chromosome examination showed the emergence of up to 24% and then 40% of cells with a normal karyotype. These findings were confirmed by whole-exome sequencing. Similar results were obtained for cells with TR18, known as Edwards syndrome. The authors suggest that introduction of human *ZSCAN4*-mRNAs into cells may have the ability to remove chromosome extracopies without affecting the remainder of the genome. They speculate that a *ZSCAN4*-mediated mechanism detects unpaired chromosomes during cell division (meiosis or mitosis) and detaches them from the rest of the replication apparatus.

An unexpected result was reported by Inoue et al. [20]. They established independent iPSC lines derived from amniotic fluid obtained from a female fetus with DS associated with polyhydroaminios at 29 weeks of gestation for the purpose of reducing amniotic fluid. Karyotypic analyses confirmed that all iPSCs contained Hsa21 trisomy in all the cell lines. They then continuously cultivated the iPSCs lines for 70 weeks. At that time, normal Hsa21 diploids were observed in 20% of the cells. The experiment was repeated several times to make sure that trisomy rescue was not due to Hsa21mosaicism. Expression counts based on gene chip analyses performed on the diploid and the TR21 iPSCs indicated that the expression levels of the genes for *DYRK1A*, *SOD1*, *ETS2*, *APP*, and *DSCR1* decreased to two-thirds in diploid iPSCs Hsa21 compared to TR21 iPSCs. This implies that the revertant cells had regained normal genic expression. A sample of the two types of iPSCs cells was differentiated into neural stem cells (NSCs) as to morphology and neural marker expression. It was observed that the expression levels of genes *APP* and *DSCR1* in TR21 NSCs were higher than in diploid NSCs.

The authors attribute the observed spontaneous reversion from trisomic cells to disomy without genetic manipulation, chemical treatment, or exposure to radiation, to mitotic chromosome nondisjunction during iPSCs long-time cultivation. Trisomic rescue may be tissue-dependent [21]. This could mean that the tissue environment of aneuploid cells is a key variable in chromosome regulation.

Although the way towards clinical applications of the above technologies may still be far off, these results obtained suggest the feasability of normalizing operations on trisomic cells at least in vitro.

### 2.2. Acting on genes

The genetic “scissor” CRISPR-Cas9 can perform precise cutting on the DNA and RNA ribbons. It is possible to remove a specific portion of a chromosome and see what happens when particular genes are removed. Enzymes that have the ability to catalyze larger DNA or RNA molecules are employed.

One may envisage removing or inactivating triplicated genes located on Hsa21. Partial corrections may already have a phenotypic effect. This is suggested by the milder phenotype characteristic of those cells of TR21 mosaicism that have a normal karyotype.

Efforts are being made to identify the particular set of genes whose overexpression determines the brain alterations observed in persons with DS. Several genes may interact in impairing neurological development. Some genes on Hsa21 may also have an impact on other genes in the genome.

A number of genes on Hsa21 show dosage effect in TR21. This means that their expression is increased (more or less 50% in general) in the cells or tissues of persons with DS. This increase can be measured by biological activity (enzymatic, for example), quantity of proteins produced, or mi-RNAs. Ait Yahia-Graison et al. [4] counted 120 genes expressed in lymphoblastoid cells derived from 10 persons with DS (3 women and 7 men) and 11 controlled individuals (4 women and 7 men). Twenty-two percent of these genes were overexpressed in DS cells in correspondence with the gene-dosage effect and 7% were amplified beyond this level. Fifteen percent were highly variable among individuals with DS, which may account at least partially for the phenotypic variability observed in the syndrome. Fifty-seven genes were found to be compensated by decreased or increased transcription, which the authors consider an indication of genetic robustness.

The overexpressed genes in TR21 include: *APP* (amyloid-bêta precursor protein), *SOD1* (superoxide dismutase-1), *DYRK1A* (dual specificity tyrosine Y-regulation kinase 1A), *EURL* (bêta-catenin signaling modulator), *CBS* (cystathionine-bêta synthase), *OLIG1* and *OLIG2* (oligodendrocyte transcription), *IFNAR* (alpha-interferon receptor), *CBR1* and *CBR2* (carbonyle reductase), *S100B* (glial function in neurons), *ERG* (regulator of hemato-immune cells), *DSCR1* (inhibitor of calcineurin-mediated signaling), *RCAN1* (calcineurin regulator), and *ETS2* (encoding a transcription factor).

However, there exists important variations in gene expression in the DS condition. As already suggested [22], it is unlikely that all genes on Hsa21 would produce equally marked biological harm when triplicated because this would have lethal consequences for the affected persons.

As suggested in DS, DS-derived iPSCs, and DS mouse models, overexpression of genes *DYRK1A*, *APP*, *EURL* involved in various cell functions and structural aspects of neurogenesis, of *OLIG1/2* responsible for myelinating cells and oligodendrocyte differentiation, and of *ERG*, and *RCAN1* affecting the central nervous system, is probably among the most noxious mechanisms in T21 brain etiopathology [23].

For example, *DYRK1A* transgenic mice exhibit neurogenesis alterations and brain and behavioral abnormalities comparable to those of human beings with T21 [24]. Thomazeau et al. [25] found that overexpression of *DYRK1A* increases the number of spines on oblique dendrites of pyramidal neurons in the pre-frontal brain of adult mice trangenic for gene *DYRK1A*. Li et al. [26] observed that perturbation of *EURL* mRNA levels in mice C57BL/6 impairs progenitor proliferation and neuronal differentiation, and reduces the dendritic spine densities of cortical neurons. These authors objectified similar features in tissue samples from human fetuses between 16-19 weeks of gestation. Chakrabarti et al. [27] established that overexpression of *OLIG1* and *OLIG2* in the forebrain of mice Ts65Dn leads to defective neurogenesis.

Manley and Anderson [28] observed that *OLIG2* gene dosage alters cerebral cortical interneuron development and contributes to cognitive disability in mice. Ishihara et al. [29] observed that *ERG* gene triplication contributes to dysregulation of the homeostatic proportion of the populations of immune cells in the embryonic brain and decreases prenatal cortical neurogenesis in a mouse model.

There are a large number of RNAs, some protein-coding and others non-coding. Hsa21 harbors four major types of micro RNAs (miRNA-99a, miRNA-125b, miRNA-155, and miRNA-802). These are short non-coding RNAs mediating post-transcriptional gene silencing, whose overproduction is involved in the etiopathology of T21. Selective inactivation of all or at least some of these RNAs could be an efficient strategy for improving the DS phenotype [30]. Other RNAs can be used to silence other genes (see [31], for an example).

Wang et al. [32] documented an association between T21 and abnormally low levels of proteins SNX27 in cells. This protein protects neurons from excess quantities of neurotransmitter glutamate. Increased production of miRNA-155, linked to the triplication of several genes on Hsa21, is correlated with a reduced amount of SNX27.

Wang et al. [32] used mice genetically modified to produce fewer proteins SNX27 (mice named SNX27+/−). These mice showed a learning and memory handicap associated with reduced synaptic recycling of glutamate in spite of a globally normal neuroanatomy.

Synapses operate in association with specific neurotransmitters. They are produced pre-synaptically and recuperated post-synaptically. Wang et al. [32] engineered a therapy at the level of the hippocampus for restoring normal levels of protein SNX27 and post-synaptic glutamate recycling. This resulted in better learning and memory in the treated mice.

Murine models are useful tools in genetic research and engineering. However, TR21 in humans is orders-of-magnitude more complex than the corresponding mouse models, especially related to cognitive functions.

Mice Ts65Dn and Ts1Cje, for example, are genetically modified to correspond to the triplication of Hsa21 in humans (see [30] for a differential analysis of various mouse models). Ts65Dn partially mimics the DS human condition including the developmental delay and memory deficit. Ts1Cje mice exhibit drastic limitations in spatial learning and present craniofacial alterations. Genes in mice orthologous to Hsa21 are distributed on Mmu chromosomes 10 (39 genes), 16 (112 genes), and 17 (19 genes). In these regions, the orthologous genes are syntenically conserved. Ts65Dn mice have genes corresponding approximately to 60% of the genes harbored by Hsa21 [33].

A recent study by Aziz et al. [34] suggest that there are important phenotypic differences between cytogenetically distinct mouse models of DS. Using the same longitudinal experimental design, they compared Ts65Dn, Ts1Cje, and a third variety of mice, Dp(16)1/Yey. This last variety, engeneered more recently, duplicates a 23.3 megabytes segment of Hsa21(119 genes).

This means that, although these mouse models are extremely valuable for the study of genotypic/phenotypic features in DS, they are incomplete. Mice with full T21, i.e., with all their genes ortholog to those on Hsa21 triplicated, can be created by complex crossing of genetic lines (for example, [35]). But they are difficult to produce, expansive, and short living.

Transgenic mice are mice whose genomes have been modified by the transfer of a gene or a chromosome from another species.

Fillat et al. [31] employed an adenovirus-associated AAV2/1-shDYRK1A as vector to carry a miRNA into the hippocampus of experimental mice Ts65Dn for reducing the expressivity of gene *DYRK1A* and in the control group a virus with a sequence that does not interfere with the gene *DYRK1A* (AAV2/1-scDyrk1A(SC). Separately, a lentivirus LY-anti-miR155-802 was used to increase the expression of another gene, *MECP2*, located on the X chromosome at the locus Xq28. In both cases, a statistically significant improvement (*p* < 0.01) was observed in the learning ability of the treated mice on the Morris aquatic labyrinth against the euploid mice.

This research suggests that a modification of genes located on other chromosomes than the 21st, for example a downregulation of *MECP2*, may also have a role in the DS phenotype. It also opens a biological way to address the reduced or suppressed function of the gene *MECP2* on the active X chromosome that is causally involved in the etiology of Rett syndrome; a degenerative condition in female children and adults characterized by serious difficulties and limitations in language, motor behavior, and intellectual capability.

Mouse chimeras are used in experimental neurology to investigate cell development. They are obtained by introduction of targeted embryonic stem cells into blastocysts (early embryonic stage) resulting in animals that have two (or more) populations of genetically distinct cells coming from different zygotes.

Xu et al. [36] observed that iPSCs derived from persons with DS overproduce *OLIG2* ventral forebrain neural progenitors, which favors excess production of sub-classes of GABAergic interneurons (neurons organizing circuits between efferent and afferent neuronal bodies). Transferred in neural chimeric mice, this causes impaired memory recognition. Short hairpin RNAs (shRNAs) were used to reverse abnormal *OLIG2* expression. This reduced interneuron production in DS iPSCs and chimeric mouse brains, which in turn reduced behavioral deficits in the latter. The implication drawn by the authors is that altered *OLIG2* expression may underlie neurodevelopmental abnormalities and cognitive defects in persons with DS.

Chakrabarti et al. [27] removed one allele of each triplicated gene within the genome of Ts65Dn mice by breeding Ts65Dn females with *OLIG1/2* double heterozygous males to normalise the dosage of genes *OLIG1* and *OLIG2* in the forebrain of the resulting pups. Returning Ts65Dn animals to disomy for *OLIG1* and *OLIG2* genes had the effect of normalising neurogenesis. This restituted a normal balance between excitatory and inhibitory neurons in the central nervous system alleviating a defect in synaptic plasticity due to the overinhibition phenotype suspected to be one of the underlying causes of cognitive deficit in Ts65Dn mice.

Ishihara et al. [29] restored the perturbed proportions of immune cells in Ts1Cje mice embryo brains through a genetic manipulation rendering these mice disomic for the *ERG* gene (but otherwise trisomic on a Ts1Cje background). The neurogenesis defects observed in Ts1Cje were reduced in the *ERG* modified mice embryos. This finding suggests that *ERG* gene triplication may contribute to a dysregulation of the proportions of immune cells in the DS embryo, which perturbates pre-natal cortical neurogenesis.

Acting on genes may not be without risk, however. The immunological systems have evolved to contrast viral aggression. It is imperative to make sure that reprogrammed cells are not confounded with infected ones and end up as targets for destruction.

Gene correction may become technically possible in DS in the middle-term, including in pre-natal stages. It should be envisaged with extreme caution as major risks exist for mothers and fetuses given the toxicity of the viruses, possible adverse immunological reactions, and the risks of tumor generation [37].

### 2.3. Acting on gene products

Besides modifying genes, it is possible to operate on the proteins and enzymes encoded by these genes.

Nakano-Kobayashi et al. [38] reported that the oral administration of a growth inducer identified in their screen of neural stem cells (NSCs), named Algernon (for “altered generation of neurons”), which they claim has an inhibitory activity against the expression of gene *DYRK1A*, rescued NSC proliferation and increased the number of newborn neurons in Ts65Dn mice derived neurospheres and in human NSCs derived from human fibroblasts with DS. When administered to pregnant Ts65Dn dams, the medication induced improved cortical formation and prevented the development of abnormal behavior in the Ts65Dn mouse offsprings. The authors suggest that ALGERNON pre-natal therapy may have the capacity of preventing structural and developmental aspects of the DS neurogenic phenotype. However, no precise information is given on the nature and chemical composition of the algernon molecular compound.

A catechin molecule named epigallocathechin 3-gallate (EGCG), a polyphenol of green tea with antioxidant properties, has generated much interest in recent years. It is a natural inhibitor of the enzyme encoded by gene *DYRK1A*.

Experiments with murine models support the efficiency of EGCG for rescuing various aspects of neurogenesis. For example, in the course of the study mentioned before, Thomazeau et al. [25] administered drinking water containing 25% green tea decaffeinated extract (0.08 mg/ml) and 25% glucose to male mice Tg189N3 (mBACtgDyrk1a) aged 4–6 months for 4 to 6 weeks. These mice with a third copy of *DYRK1A* expressed more DYRK1A brain proteins compared to wild-type littermates. Green tea extracts contained 45% EGCG. Daily doses ranged between 120 and 200 mg per kilo of weight. Thomazeau et al. observed a normalization of spine density in deep layer pyramidal cells of the prefrontal cortex associated with a rescue of long-term potentiation of synapses in memory structures. This study suggests that the origin of the morphological and functional *DYRK1A*-related deficits in the pre-frontal cortex is not merely developmental. Continued overexpression of *DYRK1A* in adult times may also be detrimental to brain pre-frontal functioning. Contrasting *DYRK1A* excessive activity also in adolescents and adults years could be justified in DS.

Hibaoui et al. [39] induced iPSCs from primary fetal skin fibroblasts obtained *post mortem* from discordant monozygotic twins, one with T21 (twin-DS) and the other normal (twin-N) in an attempt to control for the genomic background. Both the iPSCs and the NPSCs (i.e., iPSCs derived into neural cells) exhibited defects associated with changes in the architecture and density of neurons, glial cells and with the expression of genes involved in neurogenesis. In particular, a two-fold increase in DYRK1A enzymes was found in NPSCs.

Hibaoui et al. [39] reported rescuing neurogenesis impairment in these cells through *DYRK1A* inhibition using EGCG or a short-hairpin RNA silencing (shRNA); the latter one in order to exclude a simple anti-oxidant effect of EGCG.

EGCG improved the number of NPSCs derived from twin-DS-iPSCs by promoting cell proliferation and preventing apoptosis. When these NPSCs were further induced into mature neurons, *DYRK1A* inhibition through EGCC or shRNA treatment improved the expression of neuronal markers B3-TUBULIN and MAP2, an indication of improved neurogenesis.

A reduced expression of several genes, up to 30%, in NPSCs derived from the twin-DS-IPSCs, was promoted by restoring *DYRK1A* expression to near normal levels by shRNA, confirming that the *DYRK1A* gene when overexpressed is a major contributor to impaired neurogenesis in DS.

Moreover the genetic profiling of the twin-DS-iPSCs compared to the twin-N-iPSCs revealed that TR21 not only affects the expression of trisomic but also of disomic genes. As many as 96 genes related to brain functions were found to be downregulated. This finding suggests that TR21 actually determines an alteration of the whole set of RNAs resulting from gene expression, as suggested by Lyle et al. [3].

Corresponding data have been published by Guedj et al. [40]. They submitted transgenic Ts65Dn and Ts1Cje mice carrying an additional copy of gene *DYRK1A* to a diet rich in green tea polyphenols from gestation to adult age. The study included four groups of mice: wild-type and trangenic, fed either with water or green tea. Green tea infusions were given daily. The chronic polyphenol-based diet rescued major features of the transgenic phenotype as demonstrated by significant statistical differences between the groups in brain weight, brain magnetic resonance imagery, and volume of the thalamus-hypothalamus region (*p* < 0.05).

The above studies and others (see Stagni et al. [41], for a summary of additional works) suggest the interest of an EGCG strategy for improving neurogenesis in mice genetically modified or transgenic for the gene *DYRK1A*. It must be noted, however, that these studies differ from each other in several respects that may interact with the effect of EGCG: for example, length of treatment, age of the animals, dosage, administration of other green tea extracts in ill-defined combinations with EGCG (see Stagni et al. [41], for an overall discussion). This prevents solid conclusions to be drawn.

Very few studies have been conducted for testing the effect of EGCG at the human level. In a first experiment, De la Torre et al. [42] witnessed positive effects on visual recognition in a group of young adults with DS following three months of daily treatment with EGCG (green tea extracts, low in caffeine, 9 mg per kilo of body weight, administered orally). No adverse event linked to the medication was observed. However, three months after the end of treatment, the participants' performance returned to pre-intervention level.

In a second study, De la Torre et al. [43] tested the effect of a daily EGCG treatment (with a dosage identical to the preceding study), lasting for one year, coupled with a behavioral program of cognitive training. The sample included 84 adults with DS (about as many women as men) aged 16 to 34 years. They were divided into two groups: one treated with EGCG and undergoing cognitive training; the other receiving a placebo and the same cognitive training as the first group. Long-term administration of the medication in the experimental group appeared to be well tolerated.

A battery of neuropsychological tests was administered at the end of the study. Participants treated with EGCG and exposed to cognitive training showed a statistically significant superiority (*p* < 0.05) in two cognitive tasks (memory and visual recognition) and in several adaptive tasks (daily routines abilities). A retest 16 months later showed partial persistence of the effects measured at the end of the intervention.

The above data suggest that EGCG may improve brain defects in TR21 mice, genetically modified or transgenic. It is questionable, however, whether the product actually eliminates these defects. At the human level, 12 months of treatment with green tea extracts containing EGCG plus cognitive training seems to have a positive effect on cognitive performance, albeit of a modest magnitude, and this effect is partially retained after discontinuation of the treatment.

Xicota et al. [44] tested the effect of EGCG on the lipid profile of individuals with DS. It is known that many of these persons tend to have higher rates of obesity. A double-blind clinical trial compared the effect of EGCG administered during 12 months to a placebo on the body lipidic composition of 77 young adults with DS. Individuals receiving a placebo showed the expected increase over time in body weight and body mass index (ratio of body weight to squared body height). A similar increase was not observed in participants receiving an EGCG treatment. However, the group difference was statistically significant (*p* < 0.05) only for the male subjects.

An anonymous survey has been conducted by Long et al. [45] on parents' attitudes regarding the administration of green tea extracts containing EGCG. Parents who give green tea extracts to their children with DS are mostly younger, highly educated and they tend to consult scientific sources. The individuals with DS who receive green tea extracts are characterized as less severely disabled to begin with. Most caregivers who do not give green teas extracts report to be concerned about potential negative side effects and they have doubts regarding treatment effectiveness.

## 3. Early diagnosis

The DS brain starts at a disadvantage and the disadvantage is cumulative. It is essential that the attempts at improving neurogenesis and neural connectivity be conducted as early as possible in life. Major phenotypic features of DS can already be traced back to the fetal period. Early intervention implies early diagnosis of the condition.

Prenatal screening for DS is possible from the eleventh week of pregnancy through the analysis of fetal DNA fragments or mRNAs in maternal blood samples. Ninety-nine percent reliability can be reached in combining ultrasound, blood analysis, cardiac rhythm, and nuchal translucency [46]–[48].

A less expensive technique has been experimented by Shan et al. [49]. They analyzed the peptidome of urine samples of pregnant women carrying fetuses with DS and with normal karyotypes. A classification model was constructed based on candidate peptides that could differentiate fetuses with DS from controls reaching a sensitivity of 95.7% and a specificity of 70%. This suggests that a maternal urinary peptidome could offer a prospect for a noninvasive biomarker screening of fetal DS.

Amniocentesis and chorionic villus sampling remain the only fully reliable diagnosis techniques, but they are intrusive and there is a risk of a miscarriage between 0.5 and 1% [50].

Progress is being made in the development of noninvasive prenatal diagnostic methods. Asim et al. [2] have published an analysis of the advantages and disadvantages of a series of molecular methods for prenatal diagnosis of DS (e.g., cytogenetics analysis, fluorescence in situ hybridization). A noninvasive diagnostic technique has been experimented by Zbucka-Kretowska et al. [51]. They studied the expression level of miRNAs in the plasma of 198 pregnant women with fetal DS at 15–18 weeks of gestation and 12 women with uncomplicated pregnancies who delivered healthy newborns at term.

Out of 800 miRNAs analyzed by Zbucka-Kretowska et al. [51], six were upregulated and seven downregulated in plasma samples of women with fetal DS. The genes regulated by these miRNAs are involved in central nervous system development, congenital abnormalities, and heart defects. This study opens the way towards designing a panel of miRNAs for a nonintrusive technique of prenatal DS diagnosis.

Genetic corrections in the early stages of prenatal development could become possible in the future. The ultimate target is embryonic gene correction inducing normalized genetic expression in most body cells. The technology exists pending further refinements and security checks. It will leads to the development of complexes of mosaic cells, part normal, part aneuploidic, as there is no way to modify the entire embryo's line cell in the present state of gene editing technology.

It is known that neurogenesis continues in the ventricular and subventricular zones of cerebral cortex during the third trimester of pregnancy [52]. Later corrective intervention in fetuses with DS therefore could still have a therapeutic interest.

## 4. Molecular pharmacology

The neurobiological consequences of DS result in reduction of synaptic density and plasticity. Much attention has been devoted recently to the neurotransmitters. Gotti et al. [53] have published a review of studies on the alterations of brain circuits that can be identified in murine models of DS. It shows that different neurotransmission systems are downgraded or upgraded in several cerebral regions including the hippocampus, the locus coeruleus, and the frontal cortex.

Major neurotransmitter systems involved in brain functions are the cholinergic system (i.e., an excitatory neurotransmitter), the noradrenergic system (with the mostly excitatory neurotransmitter noradrenaline), the glutamate system (i.e., brain's major excitatory neurotransmitter), the GABAergic system (i.e., brain's major inhibitory neurotransmitter), and the serotoninergic system (also inhibitory).

Drugs are being developed for reducing the neurotransmission deficits caused by TR21. Interesting results have been obtained with the Ts65Dn murine model of TR21. Several substances have been shown to rescue at least partially learning and memory deficits. In some cases, attempts have been made to extent the intervention to humans with DS.

### 4.1. Cholinergic system

Inhibitory agents of acetylcholinesterase have been tested which catalyzes acetylcholine post-synaptically reducing it to its basic constituents and allowing neurons to return to a state of rest following activation.

Administration of physostigmine has been demonstrated to inhibit acetylcholinesterase and increase concentration of acetylcholine in the synaptic area in Ts65Dn mice at 4 months of age (but no longer at 10 and 16 months) and to improve learning and memory [54].

Maternal choline acetyltransferase supplementation from conception to weaning has been showed to improve spatial learning in mice derived from Ts65Dn dams and tested on a water maze at 14–18 months. Hippocampal tissues were examined for intensity of choline acetyltransferase immunoreactivity. The increased innervation produced by choline supplementation appears to improve hippocampal function [55].

At the human level, Heller et al. [56] have reported an open-label case series of six non-demented adults subjects with DS (aged 20–41 years) receiving 5 then 10 mg donepezil over 24 weeks. Despite transient side-effects expectable from cholinergic overstimulation, all subjects tolerated well the 10 mg dosage. A small but significant improvement (*p* < 0.05) in expressive language was observed at 24 weeks in four sub-tests of the Clinical Evaluation of Language Function-Revised (CELF-R). However, the small sample of subjects and the lack of a control group render the outcome of this experiment difficult to interpret.

Heller et al. [57] reported results from a 16-week pilot clinical trial on the effects of donepezil on the language of 5 children with DS aged 8 to 13 years. The drug was dosed orally at 2.5 mg once daily for 8 weeks and at 5 mg for the remaining 8 weeks. Two language measures were used: the Test of Problem Solving (TOPS) and the Clinical Evaluation of Language Fundamentals (CELF-3). Medication effects were measured by changes from the baseline to performance at weeks 8 and 16. No subject experienced serious adverse effects from the administration of the medication. T-tests yielded no significant indication of change in language performance between base line and TOPS scores at 8 and 16 weeks. However, a significant improvement (*p* < 0.003) was registered in the mean CELF-3 performance from baseline to week 16. Results from this study are also difficult to interpret owing to the lack of a control group and the small sample of subjects.

Kishnani et al. [58] tested the efficiency of donepezil in a sample of 123 young adults with DS (aged 18–35 years), who had no evidence of AD, in a 12-week randomized, double-blind, and placebo-controlled study. Experimental subjects were treated with doses of 5 mg per kilo of weight for 6 weeks and then 10 mg/kilo in the following 6 weeks. Cognitive measures included the Severe Impairment Battery (SIB), the Rivermead Behavioral Memory Test for Children, and the Clinical Evaluation of Language Fundamentals (CELF-3). Additionally the Vineland Adaptive Behavior Scales were administered. Donepezil appeared safe. However, two subjects had to be withdrawn from the double-blind phase for hypertension rated as possibly problematic by the investigators. Outcomes suggested efficacy of the drug in some but not all subjects, which the authors estimate consistent with the phenotypical variability in DS. However, improvements were also observed in the placebo group, particularly on the SIB, during the double-blind phase of the study, preventing clear-cut conclusions regarding the cognitive specificity of this molecule.

Kishnani et al. [59] assessed the efficiency and safety of donepezil with 129 children and adolescents with DS (aged 10–17 years) in a 10-week, randomized, double-blind, placebo-controlled, and multicenter study. Participants received a dose of 2.5 mg/kilo donepezil in the first part of the experiment, increased every 14 days until reaching 10 mg/kilo. Measures included the Vineland-II Adaptive Behavior Scales Parent/Caregiver Rating Form (VABS II/PCRF). The medication appeared to be well tolerated. During the double-blind phase, the VABS II/PCRF scores improved significantly (*p* < 0.001) in both the treated and control groups but with no significant difference between groups.

Rivastigmine was tested by Heller and associates [60],[61] in two open-label studies with 10 children and adolescents with DS (8 boys and 2 girls) aged 10 to 17 years. Doses at the beginning of the intervention were 1.5 mg/kilo increased to 3 mg/kilo and 4.5 mg/kilo in the following weeks. Five subjects reported no adverse events with the medication, but five others signalled transient vomiting, diarrhea, fatigue, or insomnia related to cholinergic enhancement. After 16 weeks, there were statistically significant gains in expressive language on the Test of Verbal Expression and Reasoning (TOVER; *p* < 0.02), as well as for language measures (narrative memory and immediate memory for names on the Developmental Neuropsychological Assessment Test (NEPSY; *p* < 0.02). There were also significant improvements in attention on the Leiter-R Attention Sustained Tests A and B (*p* < 0.01 and *p* < 0.02, respectively). Five participants (4 boys and 1 girl) continued the treatment for another 38 months. Longer-term use of rivastigmine appeared to have no adverse effect on overall health. A comparison of median performance change between those who continued the treatment for 38 months versus those who did not yielded no statistically significant difference. However, two subjects demonstrated important improvements in adaptive function (measured on the VABS) over the longer-term period with continued rivastigmine administration.

The results of the above studies suggest that the effects of donepezil and rivastigmine administration are not consistent across individuals with DS. Many participants show little to no gains but a small subset of individuals seem to respond positively to the cholinergic intervention.

### 4.2. Noradrenergic system

A reduction of the concentration of thyroid hormones is observed in the locus coeruleus of Ts65Dn mice from 6 weeks of age in comparison with normal mice. This can be hypothesized to reduce the supply of noradrenaline in the hippocampus. Ts65Dn mice treated with formoterol that stimulates the adrenergic receptors appear to have a more favorable neurogenesis [62].

### 4.3. Neurotransmitter glutamate

Molecules have been tested that target the receptors NMDA (N-methyl-D-aspartate) of the neurotransmitter glutamate in order to reduce an excess of activation due to this neurotransmitter. Costa et al. [63] observed that acute injections of the NMDA receptor antagonist memantine improves learning in Ts65Dn mice. Administered to young human adults with DS, in preliminary studies, memantine has shown encouraging clinical signs [64].

### 4.4. GABA system

Also in the pipeline of cognitive pharmacotherapy, is the GABA neurotransmitter system (gamma aminobutyric acid). GABA receptor antagonists picrotoxin and pentylenetetrazole have been tested by Fernandez et al. [65]. Two weeks of daily injection for each drug appears to rescue maze learning and object recognition in Ts65Dn mice. Pentylenetetrazole is controversial for possible clinical application in humans because it is known to be convulsant.

A longitudinal validation study of several cognitive scales (for example, the Leiter Performance Scale-Revised and the Clinical Evaluation of Language Fundamentals-Preschool-2) currently used to assess memory, executive function, and language in individuals with DS [66] served for optimizing trial design and endpoint selection in a clinical trial testing basmisanil, a negative allosteric modulator of GABA receptor alpha5-subtype. The trial revealed no improvement of cognitive abilities in individual with DS.

### 4.5. Serotoninergic system

A series of studies with Ts65Dn mice shows that chronic treatment with fluoxetine, an antidepressant antagonist of serotonin synaptic recapture, from postnatal days, rescues abnormalities in neurogenesis and stimulates the production of neurons and their incorporation into functional networks. Drug concentration must be carefully controlled for higher levels of fluoxetine in daily consumption may provoke seizures [62].

Guidi et al. [67] administered fluoxetine to pregnant mice Ts65Dn. After delivery, the mice whose mothers had been given fluoxetine exhibited normal brain neuronal proliferation and dendritic growth at 45 days. Fluoxetine is known as a molecule interacting with the class of enzymes histone deacetylase that catalyzes a reduction of the acetyl group thereby reducing genic expression.

Another series of biochemical agents have nootropic (nonspecific brain boosting) and antioxidant effects. They contrast continued production in the cells of biological residus of oxygen reduction (more or less 2% of the regular oxygen intake) that are harmful to the biochemistry of the body and participate to cellular aging in the long term. Overexpression of gene *SOD-1* in persons with DS leads to aggregation of hydrogen peroxide within cells and oxidative damage. Vitamin E has been tested as antioxidant therapy with positive results on memory and basal forebrain pathology in mouse models of DS [68] but no positive indication in humans with DS [69].

Piracetam increases brain oxygenation and improves GABA neurotransmission. Lobaugh et al. [70] assessed the cognitive and adaptive effects of piracetam in a double-blind study with 18 children with DS (aged 7–13 years). The experimental group received 80–100 mg/kg piracetam per day for 15 weeks. No statistically significant benefit was observed in the treated sujects in comparison with the control group in a series of tests measuring learning, attention, and memory, as well as on the adaptive scales. Treatment induced side effects of irritability and poor sleep in 7 subjects.

An antibiotic commonly used to treat acne, minocycline, seems to have neuroprotective effects and inhibit neuron apoptosis. Three months of treatment in 10-month old Ts65Dn mice significantly improved performance in cognitive tasks (*p* < 0.05) [71]. Chen et al. [72] observed that glial cells (astrocytes) supplying nutrients to the neurons and detoxifying the extracellular milieu in neutralizing excess glutamate, induced *in vitro* from stem cells derived from fibroblasts of persons with DS, do not favor *in vivo* neurogenesis when transplanted into iPSCs mice brains. Minocycline corrects this deficiency by modulating the expression of gene *S100B*, located on chromosome 21 at locus 21q22.3. This gene regulates various cellular processes including the glial function.

Broze et al. [73] tested the ability of hydroxyurea, a derivative of urea, to activate neural pathways in Ts65Dn mice. Treatment was initiated when the mice were three-month old and lasted three months. A significant improvement of memory retention of spatial information was measured in the treated animals.

On the whole, experiments with Ts65Dn mice suggest that different drugs can rescue learning and memory in cognitive tasks and in some cases improve neurogenesis. However, there is a need for standardization of the experimental protocols. In some studies, acute single doses of drugs are administered. In other studies chronic administration extends from a few weeks to several months. The ages of the treated mice vary from early pre-natal to adulthood. Sex variation is rarely carefully controlled.

A conundrum with present-day murine models concerns the non-Hsa21 ortholog genes in genetically modified mice. It is estimated that there are 50 of these genes in Ts65Dn mice. When overexpressed they should contribute to the phenotype of these animals. Also the molecular basis of drug responses must be better defined before envisaging pre-clinical and clinical trials [62].

The neurobiological gap between mice and human beings cannot be underestimated. Any generalization must be considered with maximum caution and the conclusions envisaged only hypothetically. Cognitive pharmacotherapy in DS is still in infancy and the results obtained so far with some of the drugs tested are preliminary. Drug safety and the occurrence of adverse events need to be better assessed and controlled.

## 5. Alzheimer disease

Alzheimer disease (AD) occurs more frequently and at an earlier age in persons with DS than in the general population. It affects approximately 20% of persons with DS beyond 40 years of age, 40% beyond 50 years, 80% and even more beyond 60 years. In some cases, the degenerative process is relatively slow and may occur over several years. In other cases, the pathological involution is relatively rapid. In the general population, cases of AD are found most often in persons beyond 70 years of age, although familial (hereditary) cases may have an earlier onset.

The evolution of the AD pathology is generally the same in persons with DS as in the general population except for the temporal aspects. However, there are a few differences that deserve additional investigations. For example, symptoms of depression are more pronounced and epileptic seizures more frequent in persons with DS, particularly in the early phases of the disease [74].

Doran et al. [75] submitted one man with DS when he was between 66 and 72 years to neuropsychological testing, neurological examination, amyloid *PET* imaging, and a series of related measurements. This person had partial T21 lacking triplication of the *APP* gene. The clinical phenotype was typical for DS. No dementia was ascertained on neurological examination. Post mortem neuropathological findings showed only a single neuritic plaque and neurofibrillary degeneration consistent with normal aging but not with AD. The authors suggest that APP could have an obligatory role in the clinical and neuropathological findings of AD in DS. However, the role of the *APP* gene is not clear in the evolution of normal persons developing AD, given that less than 10% of the mutations affecting this gene are responsible for early-onset AD. It could be that the *APP* gene takes part in, but is not needed for AD pathology to occur [76].

Important quantities of nontoxic amyloid-alpha proteins are produced naturally in the brain. In DS, a phenomenon not yet identified switches this production to toxic peptides amyloid-bêta 40 and 42 that cannot be dissolved in brain fluids. These peptides initiate the formation of amyloid plaques. They are tiny structures of 40 microns diameter composed of degenerated axons and dendrites, glial cells, and astrocytes centered around amyloid deposits.

The plaques aggregate around the junctions between neurons disturbing transmission. They then circumvent neuronal bodies. This seems to accelerate a tauopathy, which may start independently of the amyloid pathophysiology. It is characterized by the aggregation within the brain neurons of a protein called TAU made of helicoidal filaments of 10 nm diameter, responsible for the neurofibrillation of the cells'cytoplasm. TAU is a natural protein involved in the construction of the cell microtubules. In the AD pathology, TAU proteins become hyperphosphorylated and that destroys the neuronal tissue.

The amyloid plaques gradually invade all cortical layers, mid-brain structures, brain trunk, and cerebellum. Neurofibrillation follows a route going from entorhinal and hippocampal cortices in the medial temporal lobe to adjacent temporal cortex, frontal cortex, and then the entire brain. Alternativeley, it is also suggested that there is an early involvement of the frontal cortex and the locus coeruleus in both amyloid and tau pathology [77],[78].

The primum movens of the amyloid cascade remains unknown. A massive destruction of the Meynert nucleus, located in brain basis and supplying the departure point of the cholinergic neurons, could be at its origin [79],[80].

Prionic mechanisms are also involved in the etiology of AD. Prion proteins fold abnormally, spread through tissues and determine other proteins to unfold abnormally. This leads to infection and cerebral lesions. At least two forms of the bêta-amyloid proteins central to the pathology of AD act as prions [81]. It is suspected that TAU proteins may also display prion-like autoreplication properties [82].

Not all persons with DS seemingly develop AD. Compensatory mechanisms may operate.

It is known that other genes on chromosomes other than the 21^st^ may influence the determinism and the course of AD in aging persons without DS. A gene encoding apolipoprotein E (associated with cholesterol metabolism and intervening in a number of neurophysiological processes), located on C19, exists in three allelic variants. *APOE3* is the most frequent one in the population, followed by *APOE4* and *APOE2*. Higher dosages of *APOE3* and *APOE4* are correlated with increased and earlier risk for AD. They are associated with higher concentrations of protein amyloid-bêta, amyloid-bêta peptides, and phosphorilated TAU in the brain [83],[84].

This may be true for persons with DS as well. Raha-Chowdhury et al. [85] reported that in a cohort of persons with DS diagnosed with early dementia, the *APOE4* genotype appears to be prevalent.

In people without DS, variant *APOE2* seems to have a protective action [86]. A variant form of the *APOE3* gene, labelled *APOE3ch* (for Christchurch in New Zealand, where it was first identified) may also have a protective role (overall against the TAU pathology) particularly when present in two copies in the genome [87].

Allelic variants of the gene *SORL1* (sortilin receptor, located on C1) have been identified as additional risk factors for later AD onset [88]. When *SORL1* is overexpressed, there is an increase in brain amyloid deposits. Alterations of genes *PSEN1* (pre-senilin) on chromosome 14 and *PSEN2* on chromosome 1 also appear to be involved at various moments in the amyloid cascade; most often in the early stages in the hereditary forms of the *PSEN1* mutation, called *PSEN* E280A [89]. Vascular cholesterol may also be involved in interaction with genotype *APOE* [90].

The first molecule proposed (and approved by the US. Federal Drug Administration, FDA) for trying to control the early manifestations of AD was tacrine (tetrahydroaminoacridine), an inhibitor of acetylcholine-esterase. This increases intracerebral concentration of the neurotransmitter acetylcholine by reducing its post-synaptic degradation. However, the heavy hepatic toxicity of tacrine led the pharmaceutical firms to retire the product from the market.

Other inhibitors of acetylcholine-esterase have been developed with no particular hepatic toxicity. They include donepezil, rivastigmine, and galantamine [91] which have been approved by the FDA.

As indicated, a moderate cholinergic deficit exists in DS. It is increased in AD. In Ts65Dn mice, overexpression of the amyloid-bêta molecule has been shown to be associated with degeneration of cholinergic and noradrenergic neurons particularly in the brain hippocampal region [92]. On this basis, it make sense to try reducing the natural elimination of neurotransmitter acetylcholine with donepezil, rivastigmine, or galantamine, in chronic administration. Acetylcholine-esterase inhibitors are generally well tolerated by the patients. When side effects occur, they include nausea, vomiting, loss of appetite, and diarrhea, but are mostly transitory. So far, however, attempts to rescue cognitive functioning in persons with DS affected with AD in using acetylcholine-esterase have met only with limited success and primarily in patients at the early stages of the pathology [64].

Memantine (also approved by the FDA) is a molecule that regulates the activity of neurotransmitter glutamate. It acts on the NMDA receptors for regulating the quantity of calcium ions entering neurons in the propagation of the neural influx. Excess glutamate increases the activity of NMDA receptors causing an excess of calcium penetrating the cells and altering neural transmission. Similar side effects to those observed with cholinergic medication have been observed with chronic administration of memantine. Clinical trials with memantine have met with limited success in improving cognitive functioning in persons with DS and AD [93].

Immunotherapy addressing the first leg of the amyloid cascade has been tried with transgenic mice [94]. The strategy was to inoculate the APP protein in the vascular system, which stimulated the production of antibodies acting against the injected antigene but also against the amyloid plaques in the brain of the treated mice. A drastic reduction of the amyloid plaques was observed to be associated with cognitive improvements. Clinical trials with human subjects at the early stages of AD have been undertook. They determined a reduction of the amyloid charge in the brain of the persons treated but no improvement of the clinical symptoms.

Lithium has been tested by Matsunaga et al. [95] in an attempt to limit cognitive deterioration in persons with DS at the first stages of AD. Positive results have been reported in comparison with a control group. However, AD incipiens in DS is characterized by episodes of depression. Lithium is known to be a mood stabilizer. It is not clear whether this molecule acts mostly as an antidepressant or whether it may constitute a genuine antagonist to early cognitive deterioration in AD.

Antioxidant molecules, including nicotinamide, levocarnitine, and lipoid acid, have been tried for reducing oxidative stress, which is thought to play a role in the etiology of AD, but seemingly without clear results [96]. A long series of other molecules (e.g., phenserine, xaliproden, bapineuzumab, huperzine, intravenous hemoglobin, methylthioninium chloride, raloxifene) are in various phases of clinical trials [97]. They may lead to new pharmacological options for the treatment of AD.

Among the primary suspects in the etiopathology of AD, one finds the gene *DYRK1A*. Overexpression of this gene is thought to be one of the major culprits of the hyperphosphorylation of the TAU protein leading to the neurofibrillation of neuronal bodies.

Normalizing *DYRK1A* dosage by breeding Ts65Dn mice with a triplication of this gene with mice trisomic for the same DNA segment but without the gene *DYRK1A*, yielded Ts65Dn mice with normal dosage of this gene and a normal concentration of protein APP in cerebral cortex, hippocampus, and cerebellum [98].

Kawakubo et al. [99] treated fibroblasts obtained from persons with DS and AD pathology with harmine, an inhibitor of protein DYRK1A. Results indicated an important increase in the concentration of the enzyme neprilysin and correlatively a decrease in the concentration of DYRK1A proteins in the fibroblasts.

Accumulation of amyloid plaques in people's brains intensifies some 5 to 20 years before a significant cognitive decline is observed. Global cortical atrophy, increased concentration of TAU proteins, and neurofibrillation of the cells' cytoplasm can be objectified 1 to 5 years before a diagnosis of AD is warranted.

Current work has focused on validating a list of biological markers of early AD. It includes:

(1) Concentration of proteins APP and TAU in blood plasma [100]–[102];

(2) Blood expression of *MTRNR2L12*, a gene located on C3 (at 3q11.2), almost identical to the mitochondrial gene *MT-RNR2*, which encodes the micropeptide HUMANIN considered to be a protective factor in familial AD [103];

(3) Blood plasma neurofilament light chains [104],[105];

(4) Levels of amyloid-bêta peptides and phosphorilated TAU in neuron-derived exosomes (small extracellular vesicles secreted by the cells) [106].

Blood tests could predict onset of AD in normally aging and people with DS up to 10 years in advance in combining measures of amyloid-bêta proteins, proteins IRS-1 (involved in insulin signaling in the brain and commonly defective in people with AD), the presence of genetic variants *APOE3* and *APOE4* in blood plasma, and differences in miRNAs levels. The question is still pending, however, because the proportion of amyloid-bêta proteins in blood although correlating positively with the presence of amyloid plaques in the brain, does not ineluctabily means beginning AD. A proportion of aging people have them without developing the pathology.

Neurological examination by transcranial magnetic stimulation offers a promise for revealing synaptic dysfunctions linked to AD and predicting cognitive decline in the early phases of the disease [107].

Assuming the validity of the amyloid cascade hypothesis, a genuine curative strategy for AD is to prevent accumulation of the amyloid plaques, proliferation of TAU proteins, and neurofibrillation of neurons'cytoplasm. A number of molecules and drugs are being tested that may prove efficient, such as inhibitors of the APP protein, vaccines and antibodies, inhibitors of the TAU protein, as well as a number of other biochemical agents thought to be able to boost brain defenses against APP and TAU toxicity.

Nawa et al. [108] derived dermal fibroblasts from patients with DS. The cells showed a severe limitation of proliferation and signs of premature senescence accompanied by perturbation of homeostasis leading to accumulation of protein aggregates. They treated these cells with sodium 4-phenylbutyrate (4-PBA), a drug used to regulate urea cycle disorders, and observed a decrease in the protein aggregates of the fibroblasts.

Studies involving protein VPS35 (vacuolar sorting 35) that has the capability of altering the amyloid preprotein/amyloid-bêta metabolism, are also relevant. Li et al. [109] observed that triple transgenic mice (3xTg) overexpressing VPS35 exhibit better spatial learning and short-term memory compared to control animals. This improvement is associated with a significant reduction of amyloid-bêta levels and TAU phosphorylation. *In vitro* studies revealed reduced synaptic pathology and neuroinflammation.

Vagnozzi et al. [110] reported that an enzyme labelled TPT-172 can induce higher levels of protein VPS35. *In vitro* studies show that overexpression of VPS35 leads to a reduction of critical TAU levels in neurons. In contrast, silencing gene VPS35 is associated with an accumulation of TAU proteins. *In vivo* experiments with a transgenic mouse model of TAU pathology show that downregulation of VPS35 leads to an increase in the density of TAU proteins and a reduction of synaptic integrity. It appears that active cathepsin D encoded by gene *CTSD* on chromosome 4 is the agent mediating the VPS35 effect on TAU accumulation due to its role in degrading toxic proteins in the brain.

These latter studies open new perspectives for biochemically contrasting the second leg of the amyloid-cascade, i.e., the one concerned with TAU toxicity and neurofibrillation.

## 6. Conclusion

Important developments are taking place in the field of DS with a perspective of improvement in the life and cognitive functioning of persons affected with the condition. Genetic therapy is still largely experimental and mostly restricted to *in vitro* manipulations. It will require several additional experimental studies before being in capacity to be assessed pre-clinically and tried clinically.

Cognitive pharmacotherapy is a very active field not the least regarding intellectual disabilities and DS. Conclusive outcomes have been relatively rare so far. However, a number of molecules are in the research pipeline. Some of them could prove effective as adjunct treatments for boosting brain development and neurotransmission in persons with DS in particular. However, short- and longer-term negative secondary effects need to be controlled and the drugs possible toxicity further assessed.

Biological research on Alzheimer disease has identified a significant part of the causal chain leading to brain degeneration. However, pharmacological treatments have not demonstrated particular effectiveness except in a limited way at the early stages of the illness.

Further research on AD in aging persons without DS will benefit to persons with DS and vice versa. AD represents an important area of biological and pharmacological research and for good reasons. In the United States alone, at present time, there are 4.7 million persons diagnosed with AD. It is estimated that 20% of the persons aged beyond 80 years are affected with AD and 40% beyond 90 years. An increase is expected in coming decades associated with possible further gains in average life expectancy. In this respect, it is relevant to mention that recent statistics in the United Kingdom suggest a higher prevalence of AD in women (65% of the cases), possibly linked to longer life expectancy; presently 72 years 8 months in women versuss 68 years 4 months for men (according to the United Nations World Population Project, revision 2015; *The Guardian*, July 17, 2019).
